# Effect of Adding Bentonite to Porous Silica via the Sol–Gel Method

**DOI:** 10.1021/acsomega.3c08832

**Published:** 2024-02-21

**Authors:** Ryoko Suzuki

**Affiliations:** Materials & Research Laboratory, Advanced Technology Research & Development Division, Nikon Corporation, 1-10-1 Asamizodai, Minami-ku, Sagamihara, Kanagawa 252-0328, Japan

## Abstract

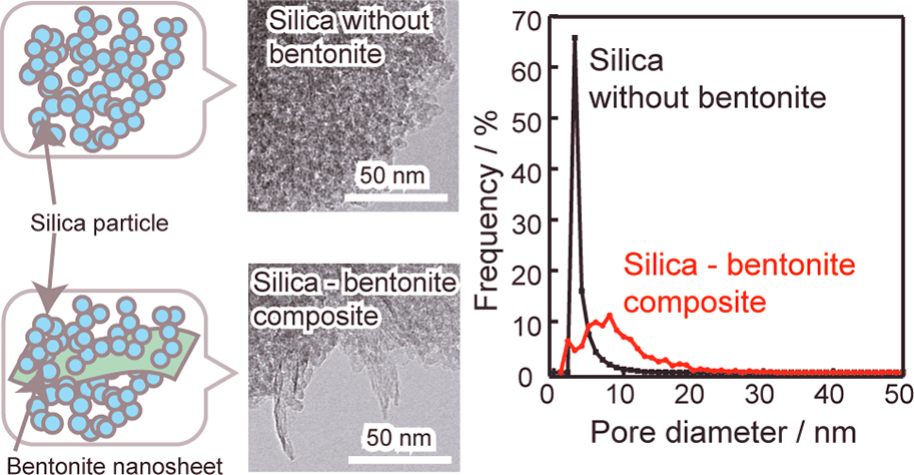

The control of specific surface area and pore size of porous materials is essential for applications such as optics, medicine, and food technology. Here, the interspace between nanomaterials such as nanoparticles and nanosheets was studied. Nanoparticle–nanosheet interspaces were formed by incorporating bentonite nanosheets to the preparation of porous silica by the sol–gel method. The product had micropore and mesopores, which originated from internanoparticle space and nanoparticle–nanosheet spaces, respectively. These two types of pores had not only different sizes but also different aspect ratios. Time-domain nuclear magnetic resonance evaluation of the bentonite dispersion revealed that the dispersion state of bentonite in water prior to composite fabrication affected the formation of the pore structure. The pore size distribution could be easily changed by adding two-dimensional and flexible nanosheets owing to the change in the physical properties of the product. The silica-bentonite composite had a significantly larger specific surface area and pore volume than porous silica without bentonite. Water vapor adsorption measurements showed that the composite exhibited a larger maximum adsorption in comparison to porous silica. Therefore, a large improvement in the physical properties can be achieved by combining nanomaterials with different geometries.

## 1 Introduction

Porous materials with high surface areas and porosities are used in various fields such as environmental science,^1^ catalyst chemistry,^2^ and optics.^3^ The required pore sizes and their distribution in porous materials depend on their applications. For example, optically transparent materials require several tens of nanometers or smaller in diameter to reduce scattering.^4,5^ For drug delivery, the pore size must match the size of the target drug and release behavior.^6^ The control of the pore size of both mesopores and micropores allows the design of moisture absorbent materials that can be applied to desiccant air conditioning systems.^7^ Therefore, the technology for controlling the pore size and distribution is critical for the application of porous materials.

Numerous materials contain artificially formed pores. Porous materials with various compositions have been prepared using the sol–gel,^8^ soft-template,^9^ hard-template,^10^ phase separation methods,^11^ and formation of pillars into layered materials.^12,13^

The structure, preparation method, and application of porous materials prepared using the sol–gel method under basic conditions have been extensively reported.^14−18^ Porous materials prepared via the sol–gel method consist of cross-linked primary particles, and their pores represent interparticle spaces. Thus, the space created by the accumulation of particles is utilized. However, there is no reported study about the size and shape of intentionally formed pores by incorporating nanomaterials into the sol–gel process.

This study aims to create pores by adding different nanomaterials to porous materials prepared by using the sol–gel method under basic conditions. The pore size distribution of products prepared by a simple sol–gel method is modified by forming particle–nanomaterial spaces in addition to the interparticle spaces obtained via the sol–gel method. Nanosheets were selected as additional nanomaterial. Nanosheets are two-dimensional materials with the highest shape anisotropy among the nanomaterials. Therefore, they were expected to form pores that were different from the interparticle spaces by interacting with zero-dimensional nanoparticles, which constitute the porous material derived from the sol–gel method.

Silica was selected as the porous matrix because porous silica is commonly available and the reaction rate can be easily controlled. In addition, exfoliated bentonite nanosheets were used as they are readily available and are unlikely to be separated from the porous SiO_2_ matrix.

## 2 Methods

### 2.1 Materials

Tetraethyl orthosilicate (TEOS; Tokyo Chemical Industry Co., Ltd.) was used as the silica source. An aqueous phosphoric acid solution (89 wt %, Tokyo Chemical Industry Co., Ltd.) was used as the catalyst for hydrolysis. An aqueous ammonia solution (29 wt %, FUJIFILM Wako Chemical Corporation) was used as the catalyst for the sol–gel reaction. Moreover, bentonite in the form of Kunipia-F (Kunimine Industries, Co., Ltd.) was used as a filler in porous silica.

### 2.2 Experimental Procedure

The samples were prepared as follows. Bentonite (0.5 g) was added to water (25 mL), and the mixture was stirred for 4 h at room temperature. The exfoliation conditions were similar to those described previously.^19^ In another bottle, phosphoric acid (41.43 μL) was dissolved in water (25 mL). TEOS (28.35 mL) was added to the phosphoric acid solution and stirred for 40 min at room temperature to hydrolyze TEOS. Immediately before mixing the above-mentioned liquids, 95.6 μL of ammonia–water was added to the bentonite–water dispersion. The two liquids were mixed and stirred until homogeneous and subsequently allowed to stand at room temperature for gelation. The gel was aged for 1 day at 70 °C and then dried at 70 °C in an oven. Silica-bentonite composite powder was obtained by crushing the dried gel. Similarly, silica without bentonite and a silica-bentonite composite with varying stirring times of bentonite in water were prepared.

### 2.3 Analysis

X-ray diffraction (XRD) analysis was performed by using a SmartLab diffractometer (Rigaku Corporation). Scanning electron microscopy (SEM) observations were conducted by using an SU9000 microscope (Hitachi High-Tech Corporation). Transmission electron microscopy (TEM) and energy dispersive X-ray spectroscopy (EDS) was performed using a JEM-2100F microscope (JEOL, Ltd.). Nitrogen and water vapor adsorption isotherms were recorded by using a BELSORP MAX analyzer (MicrotracBEL Corp.). Before the measurement of both nitrogen and water adsorption isotherms, samples were heated at 160 °C for 3 h under a vacuum. Measurement was conducted at 77 and 298 K for nitrogen and water. Surface areas and pore diameters were calculated by Brunauer−Emmett−Teller (BET) method in the relative pressure region *p*/*p*_0_ = 0.1–0.3. Total pore volumes were determined from the uptake at *p*/*p*_0_ = 0.99. Water used for measurements was pretreated with three freeze–pump–thaw cycles. Three-dimensional TEM (3D-TEM) observations were conducted by using a JEM-F200 microscope (JEOL, Ltd.). The absorbance was measured by using a Hitachi U-3900 spectrophotometer (Hitachi High-Tech Corporation). Furthermore, time-domain nuclear magnetic resonance (TD-NMR) measurement was carried by using a MagnoMeter XRS NMR spectrometer (Mageleka Inc.), operating at 12.5 MHz. For *T*_2_ measurements, the Carr–Purcell–Meiboom–Gill pulse sequence was applied. The 90 and 180° pulse durations were 7 and 14 ms, respectively. The number of scans per sample, interpulse spacing (τ), and recycle delay were 4, 0.5, and 11000 ms, respectively. The measurement temperature was maintained at 25 °C by using an external temperature control unit.

## 3 Results and Discussion

### 3.1 Structure of the Silica-Bentonite Composite

**Figure 1:**
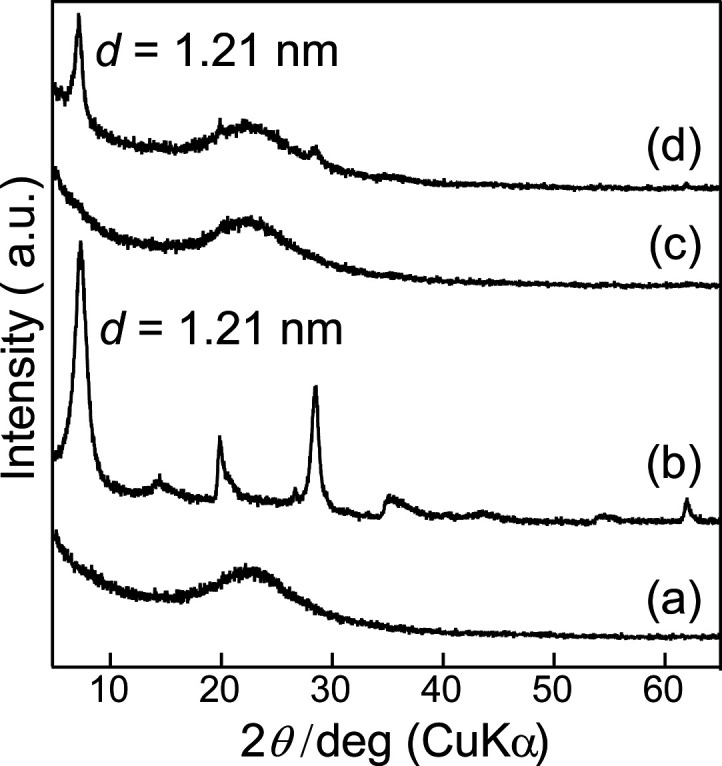
XRD patterns of (a) silica without bentonite, (b) bentonite, (c) silica-bentonite composite, and (d) a mixture of (a) and (b) with the same composition as that of (c).

Figure 1 shows the XRD patterns of the samples. Silica without bentonite does not exhibit a definite peak, indicating its amorphous structure (Figure 1a). As shown in Figure 1b, bentonite displays peaks at *d* = 1.21 nm, assigned to its layered structure.^20^ In the silica-bentonite composite (Figure 1c), no peaks assigned to the layered structure are observed. However, bentonite peaks are observed at *d* = 1.21 nm in a mixture of silica and bentonite with the same composition as that of the silica-bentonite composite (Figure 1d). These results indicate that bentonite in the silica-bentonite composite was almost exfoliated and did not maintain a layered structure. The layered structure of bentonite was considered to disappear while being stirred in water and composited with silica.

**Figure 2:**
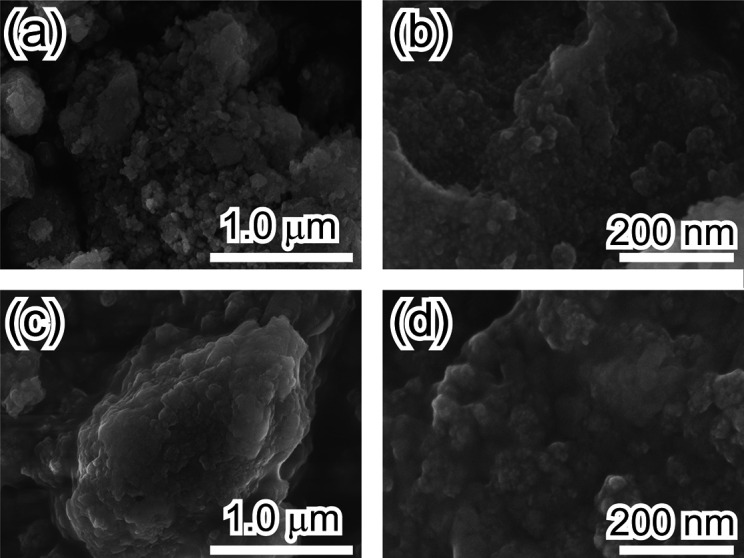
(a) Low- and (b) high-magnification SEM images of silica without bentonite. (c) Low- and (d) high-magnification SEM images of the silica-bentonite composite.

Figure 2 shows SEM images of the samples. Silica without bentonite is an irregularly shaped porous material (Figure 2a,b) and consists of connected SiO_2_ particles, as shown in Figure 2b. Similar morphologies are observed in the silica-bentonite composite. (Figure 2c,d) Particularly, a porous structure similar to that of silica without bentonite is observed in the silica-bentonite composite. As shown in Figure 2c,d, bentonite in the silica-bentonite composite appears to be almost entirely covered with silica.

**Figure 3:**
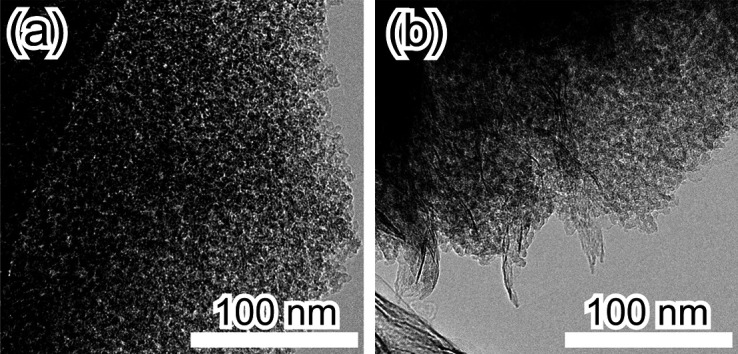
TEM images of (a) silica without bentonite and (b) the silica-bentonite composite.

Figure 3 shows the TEM images of silica without bentonite and the silica-bentonite composite. In both images, an aggregated structure with particles of several nanometers is observed. Figure 3b reveals a flaky material protruding from the interior toward the exterior of the porous structure. EDX measurements of the porous silica matrix and flaky material were performed (Figure S1). The porous silica matrix was composed of Si and O, whereas the flaky material was composed of Si, Al, and O and was identified as the bentonite component. The flexible bentonite exfoliated in water was assumed to be incorporated into the porous SiO_2_ gel as a flaky material.

### 3.2 Investigation of Pores in the Silica-Bentonite Composite

**Figure 4:**
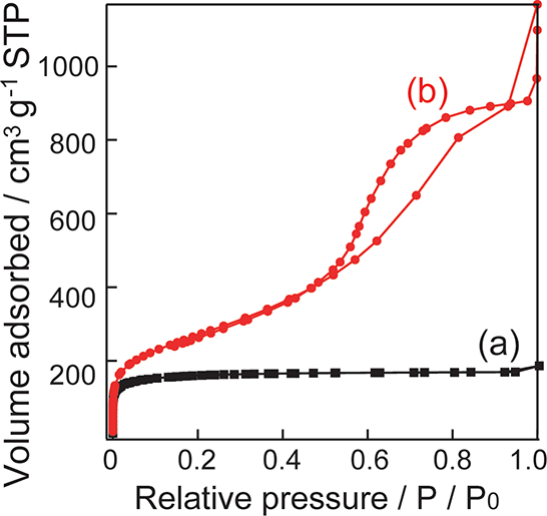
N_2_ adsorption isotherms of (a) silica without bentonite and (b) a silica-bentonite composite.

Figure 4 shows the N_2_ adsorption isotherms. Silica without bentonite exhibits a type I adsorption isotherm (Figure 4a), and BET analysis revealed a pore diameter of 2.6 nm and a specific surface area of 440 m^2^ g^–1^. However, the silica-bentonite composite exhibits a type IV adsorption isotherm (Figure 4b), and BET analysis revealed a pore diameter of 7.3 nm and a specific surface area of 958 m^2^ g^–1^. The addition of bentonite increased the pore diameter and specific surface area. Based on the SEM and TEM images, the porous silica region displays a similar structure in both samples. The increase in the pore diameter and specific surface area was attributed to the structure near bentonite incorporated into the porous silica matrix. Figure S2 shows N_2_ adsorption isotherms of bentonite and a powder of a dried bentonite aqueous dispersion. Both showed similar results in terms of relative surface area and pore diameter (Table S1).

**Figure 5:**
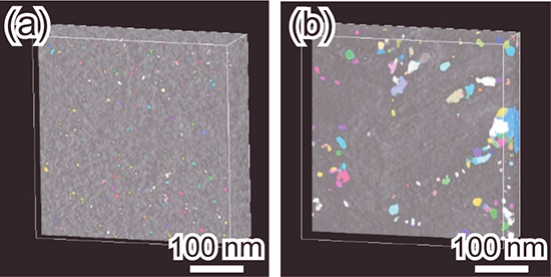
High-angle annular dark-field scanning transmission electron microscopy images obtained from 3D-TEM observations of (a) silica without bentonite and (b) the silica-bentonite composite.

Furthermore, 3D-TEM observations were conducted to investigate the shape and size distribution of the pores and the porosity of the samples (Figure 5). The porosities of silica without bentonite and the silica-bentonite composite were found to be 3 and 6 vol %, respectively. The composite has a larger pore size, specific surface area, and porosity in comparison to silica without bentonite. These results indicate that the area near the bentonite in the silica-bentonite composite has a porous structure with a thinner framework, which originated from bentonite nanosheets, compared with the porous silica matrix. Silica without bentonite has small pores (colored parts) that are distributed throughout the entire area (Figure 5a). The silica-bentonite composite also contains small pores distributed throughout the entire area and large pores distributed in various locations (Figure 5b). Large pores are observed near the bright-contrast region in the 3D-TEM image. A clear image was obtained in another region of a similar sample, and EDX analysis was performed (Figure S3). The bright-contrast region contains more Al than the dark-contrast region and consists of Al, Si, and O. Therefore, the bright-contrast regions are attributed to bentonite, which is the only raw material containing Al.

**Figure 6:**
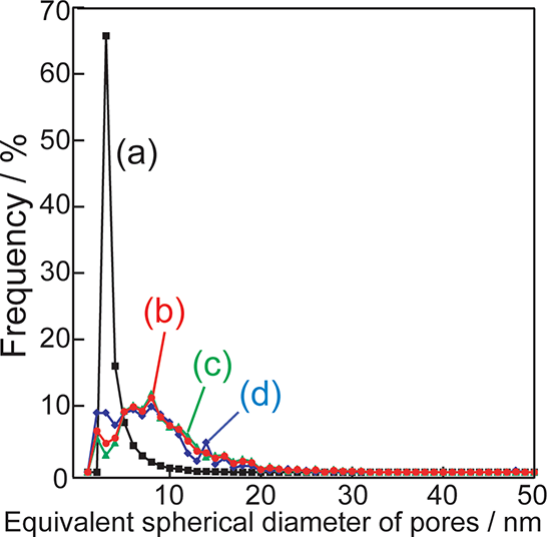
Pore size distribution of (a) silica without bentonite, (b) silica-bentonite composite, (c) area near bentonite, and (d) area away from bentonite in (b).

Figure 6 shows the pore size distribution calculated based on the 3D-TEM images in Figure 5. Silica without bentonite contains narrowly distributed pores with a peak at approximately 2 nm (Figure 6a). The silica-bentonite composite contains broadly distributed pores with a peak in the 20 nm range (Figure 6b). Thus, the addition of bentonite during the preparation of porous silica increases the pore size. This tendency is consistent with the results of the BET analysis using N_2_ adsorption measurements. Furthermore, the pore size distribution was obtained by approximately specifying the area near (Figure 6c) and away from (Figure 6d) the bentonite region. A lower number of small pores and a slightly higher number of large pores are observed in Figure 6c than in Figure 6b. In contrast, a higher number of small pores and a lower number of large pores are observed in Figure 6d than in Figure 6b.

**Figure 7:**
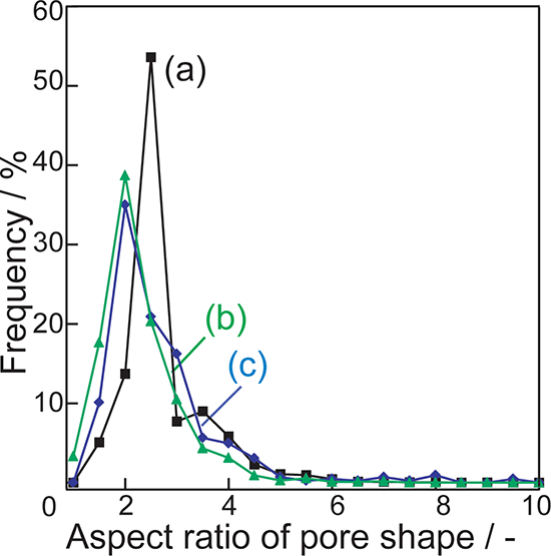
Aspect ratios of pores in (a) silica without bentonite and areas (b) near bentonite and (c) away from bentonite in the silica-bentonite composite.

Figure 7 presents the aspect ratios of the pore shapes. In silica without bentonite, a distribution with a large peak at an aspect ratio of 2.5 is observed (Figure 7a). Overall, the silica-bentonite composite (Figure 7b,c) has a smaller aspect ratio distribution than that of silica without bentonite. The area near bentonite in the silica-bentonite composite (Figure 7b) contains a few pores with large aspect ratios, whereas the area away from bentonite in the composite (Figure 7c) contains more pores with aspect ratios similar to those of silica without bentonite (Figure 7a).

At present, it is difficult to estimate and discuss the aspect ratio of the pores between nanomaterials. However, a simulation of the structure of the silica colloid in the dispersion and the morphology of the bentonite nanosheets may allow the determination of the aspect ratio of the pores by simulating^21^ the structure of the accumulation. This is a subject for future work.

### 3.3 Effect of Stirring Time of Bentonite in Water

The effect of the stirring time of bentonite in water during the composite fabrication on the physical properties of the product was investigated. Bentonite swells from the stacked structure by the introduction of water molecules into the interlayer space and exfoliates when the distance between the layers is sufficiently large. Therefore, the stirring time affects the exfoliation state of bentonite, which could be an important factor in the formation of pores.

**Figure 8:**
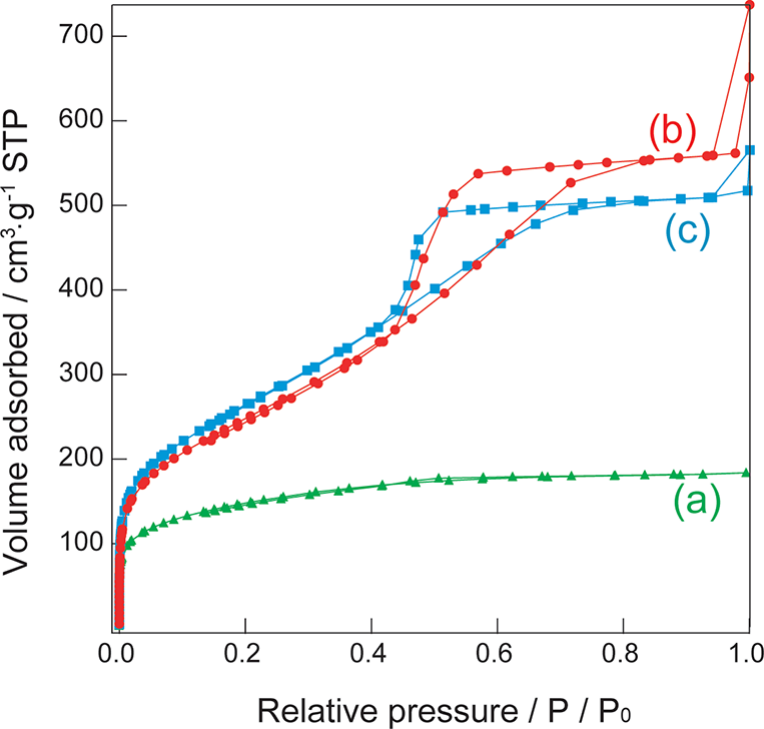
N_2_ adsorption isotherms of the silica-bentonite composite with stirring time of bentonite (a) 1, (b) 4, and (c) 24 h.

Figure 8 shows the N_2_ adsorption isotherms of silica-bentonite composite prepared by changing the stirring time to 1, 4, and 24 h. In the case of the stirring time of 1 h, type I adsorption isotherms similar to those of silica without bentonite (Figure 4a) were obtained. It was assumed that the bentonite-derived mesopores were not formed. Type IV adsorption isotherms were obtained for samples with stirring times of 4 and 24 h (Figure 8b,c), suggesting that the stirring of bentonite is important for the formation of mesopores.

The dispersion state of bentonite in water was also evaluated by TD-NMR. The *T*_2_ decay curves were separated into two components, indicating the presence of water molecules in two states. The time variation of the hydrophilicity parameter, *R*_sp_, calculated from the relaxation time^22−24^ of each water molecule and the ratio of two water molecules amount are shown in Figure S4a,b. The main components were present in large ratios, and *R*_sp_ increased by up to about 10 min after the addition of water. On the other hand, a small ratio of the subcomponent was always larger than the *R*_sp_ of the main component. The maximum value of *R*_sp_ of the secondary component/main component was reached at around 100 min.

The main component is estimated to be a large amount of water as a dispersant present in the dispersion, and its time variation is considered to indicate the deflocculation of bentonite particles. On the other hand, water of the subcomponent is adsorbed on the surface of the bentonite nanosheet or introduced into the interlayer, significantly reducing its mobility. After 100 min, when the ratio of the subcomponent began to increase, the bentonite particles are fully deflocculated, the interlayer is sufficiently swollen, and exfoliation is in progress. The number of bentonite nanosheets was sufficiently increased by prolonged stirring of the bentonite in water, resulting in a change in the pore structure of the obtained silica-bentonite composite.

**Scheme 1:**
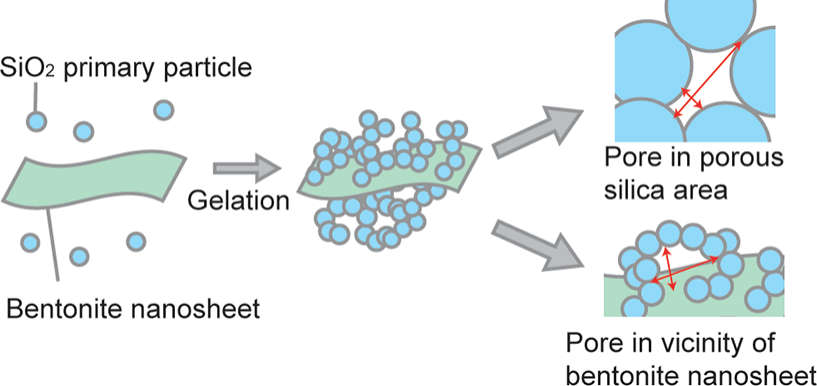
Pore Formation in the Silica-Bentonite Composite

Based on these results, the addition of bentonite during the preparation of porous silica formed pores in the vicinity of bentonite. These pores had unique sizes and shapes that differed from those formed via the sol–gel method. The pores formed via the sol–gel method could be regarded as interspaces between small silica particles and were expected to have a pointed shape with a high aspect ratio. However, in the presence of flexible bentonite nanosheets, which prevented electrostatic restacking by silica particles, low-aspect-ratio pores could be formed by the surrounding bentonite nanosheets with small silica particles formed via the sol–gel method (Scheme 1). The presence of these pores possibly contributed to the high porosity and specific surface area of the composite.

### 3.4 Evaluation of Adsorption Performance of Porous Materials

**Figure 9:**
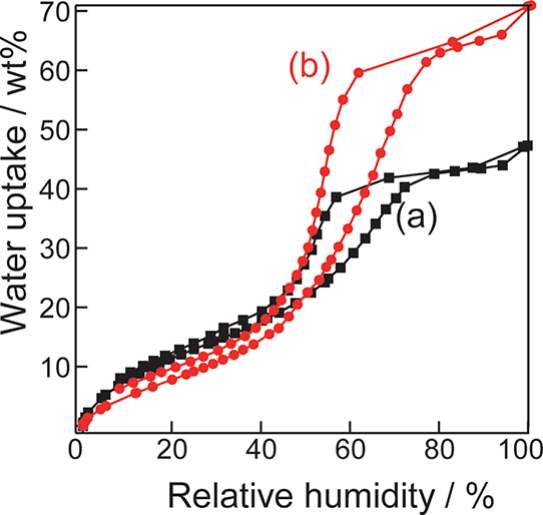
Water vapor adsorption isotherms of (a) silica without bentonite and (b) the silica-bentonite composite.

Figure 9 shows the results of the water vapor adsorption measurements. The maximum amount of water vapor adsorption is greater for the silica-bentonite composite (Figure 9b) than that for silica without bentonite (Figure 9a). This result is consistent with the trend of the pore volume (0.3 and 1.7 cm^3^ g^–1^, respectively) obtained from BET analysis using N_2_ adsorption measurements. In a previous report, porous materials with various pore sizes were compared in terms of water vapor adsorption. When RH < 10%, monolayer adsorption proceeded.^25−27^ Porous materials with mesopores adsorb less at low pressure due to monolayer adsorption and more at RH = 0.3–0.8 due to multilayer adsorption. In the case of the silica-bentonite composite prepared in this study, the micropores of the porous silica and mesopores derived from bentonite are retained and the specific surface area is large. Thus, it showed similar adsorption behavior to silica without bentonite in the low-pressure region and higher water vapor adsorption in the medium-pressure region and above. Thus, the addition of bentonite during the preparation of porous silica can improve the water vapor adsorption capacity.

**Figure 10:**
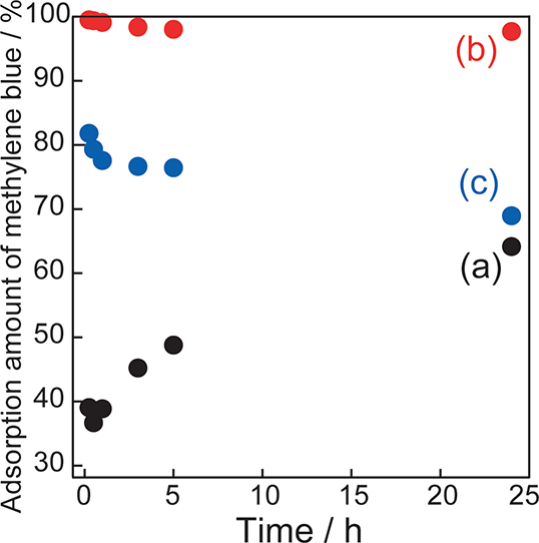
Amounts of methylene blue adsorbed on (a) silica without bentonite, (b) porous silica-bentonite composites, and (c) bentonite.

The cation-adsorption capacities of the prepared porous silica and porous silica-bentonite composites were evaluated by using a methylene blue solution. Samples (0.1 g) of the above-mentioned materials were added to a 0.01 mM methylene blue solution (100 mL) and stirred. Subsequently, the reacted liquid was filtered using a polytetrafluoroethylene syringe filter that did not adsorb methylene blue at 0.25–24 h. The resulting filtrate was used for absorbance measurements, and the amount of adsorbed methylene blue was calculated from the absorbance at 664 nm.^28^Figure 10 shows the amounts of methylene blue adsorbed by porous silica, silica-bentonite composite, and bentonite at 0.25–24 h. The amount of methylene blue adsorbed on silica without bentonite increased with time (from 0.25 to 24 h), and approximately 64.2% of methylene blue was adsorbed at 24 h. The silica-bentonite composite exhibited an extremely high adsorption rate of 99.5% in 0.25 h. After 24 h, the adsorption rate was still 97.7%, although it demonstrated a slightly decreasing trend. For bentonite, the adsorption rate decreased from 81.8% at 0.25 h to 69.0% at 24 h. The silica-bentonite composite showed the highest methylene blue adsorption over the entire period from 0.25 to 24 h. Therefore, the addition of bentonite to porous silica improved the adsorption capacity of the cations. Assuming that TEOS is completely converted to SiO_2_ in the silica-bentonite composite, the porous silica content is 93.9 wt %, and the bentonite content is 6.1 wt %. The adsorption rate of the silica-bentonite composite is larger than the sum of the adsorption rates of silica without bentonite and bentonite at each time point multiplied by the above-mentioned weight fractions. This is attributed to the increase in the specific surface area resulting from the addition of exfoliated bentonite.

The rate of methylene blue adsorption on silica without bentonite increased with time, suggesting that methylene blue was gradually adsorbed on the surface of the silica without bentonite. In contrast, the rate of methylene blue adsorption on bentonite decreased over time. This tendency was also observed in the silica-bentonite composite, indicating that the bentonite surface was exposed in the silica-bentonite composite and that methylene blue was adsorbed on both the porous silica and bentonite surfaces. However, the slight decrease in the adsorption rate of methylene blue is possibly related to the small fraction of bentonite in the silica-bentonite composite and the increase in the adsorption rate of methylene blue on porous silica with time.

## 4 Conclusions

The pore size distribution of porous silica was modified, and the specific surface area was successfully increased by adding bentonite during the synthesis of porous silica via the sol–gel method. The adsorption capacities for water vapor and cations were improved. Moreover, the addition of bentonite changed the pore shape of the porous silica. The coexistence of nanosheets in a porous material composed of cross-linked nanoparticles is considered to form a particle–sheet space that is different from the interparticle space. Furthermore, the pore structure can be controlled using nanoparticles and nanosheets as building blocks, compositing them on a nanoorder scale, and forming porous materials. Conventional nanocomposites are typically investigated in terms of controlling or improving their physical, chemical, or mechanical properties. However, their structural properties can be improved by focusing on the morphology of the constituent building blocks.

## Supporting Information Available

TEM-EDX, N_2_ adsorption isotherms and analysis of them by the BET method, HAADF-STEM-EDX, and TD-NMR results (PDF)
