# Supplementary material for: Effect of Adding Bentonite to Porous Silica via the Sol–Gel Method
Source: ACS Omega. 2024 Feb 21;9(9):10577–82. doi: 10.1021/acsomega.3c08832 (PMC10918678; doi:10.1021/acsomega.3c08832)
Supplement: Supplementary file 1 — ao3c08832_si_001.pdf [file ao3c08832_si_001.pdf]

Supporting information

Effect of Adding Bentonite to Porous Silica via the Sol–Gel Method

Ryoko Suzuki

Materials & Research Laboratory, Advanced Technology Research & Development Division, Nikon Corporation, 1-10-1 Asamizodai, Minami-ku, Sagamihara, 252-0328, Japan.

E-mail : Ryoko.Suzuki@nikon.com

Contents

A. TEM-EDX analysis of silica without bentonite and silica–bentonite composite

B. N_2 adsorption isotherms of bentonite and powder of dried bentonite aqueous dispersion.

C. Analysis of N_2 adsorption isotherm by BET method

D. HAADF-STEM-EDX analysis of silica–bentonite composite

E. Time dependence of R_{sp} value of main component and subcomponent and ratio of subcomponent and main component

A. TEM-EDX analysis of silica without bentonite and silica–bentonite composite

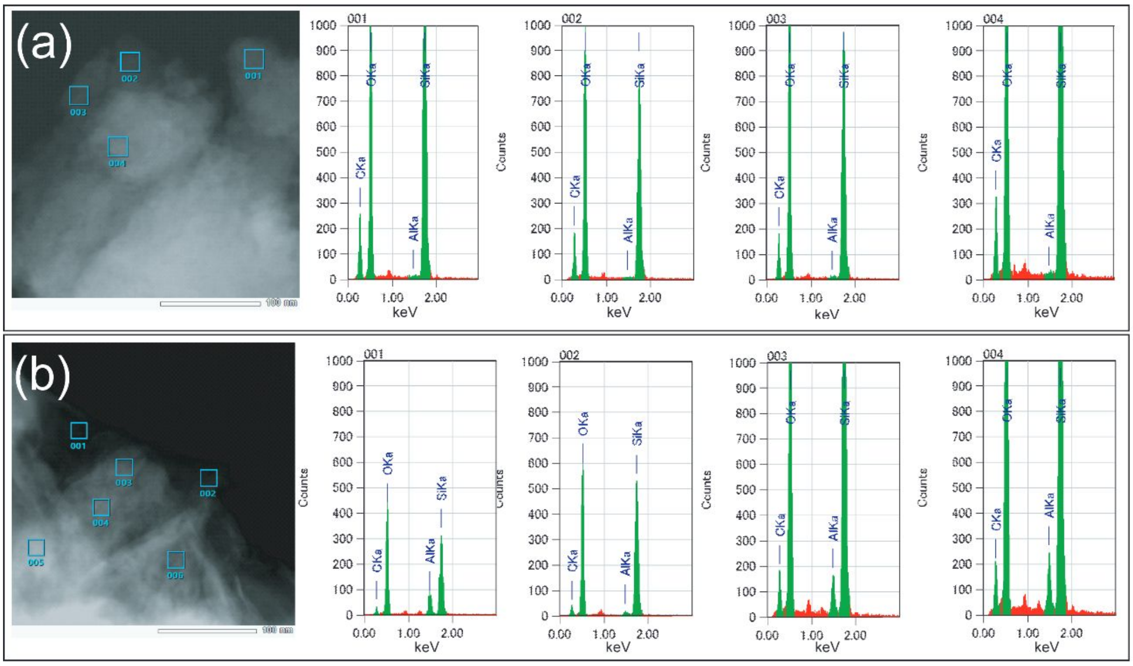

Figure S1. TEM–EDX analysis of (a) silica without bentonite and (b) silica–bentonite composite.

B. N_2 adsorption isotherms of bentonite and powder of dried bentonite aqueous dispersion.

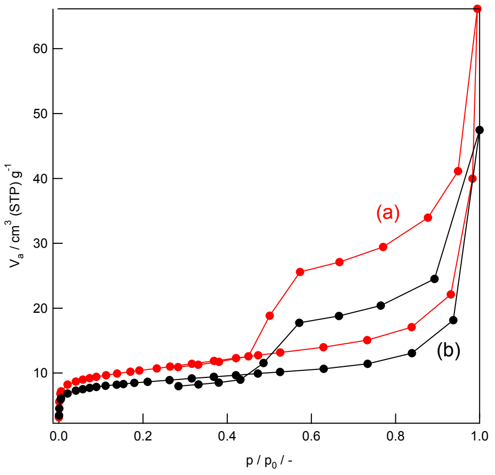

Figure S2. N_2 adsorption isotherms of (a) bentonite and (b) powder of dried bentonite aqueous dispersion.

C. Analysis of N_2 adsorption isotherm by BET method.

Table S1 Analysis of N_2 adsorption isotherm by BET method.

	Relative surface area m^2 / g	Pore diameter / nm
Bentonite	29.6	11.9
Powder of dried bentonite aqueous dispersion	25.2	10.6

D. HAADF-STEM-EDX analysis of silica–bentonite composite

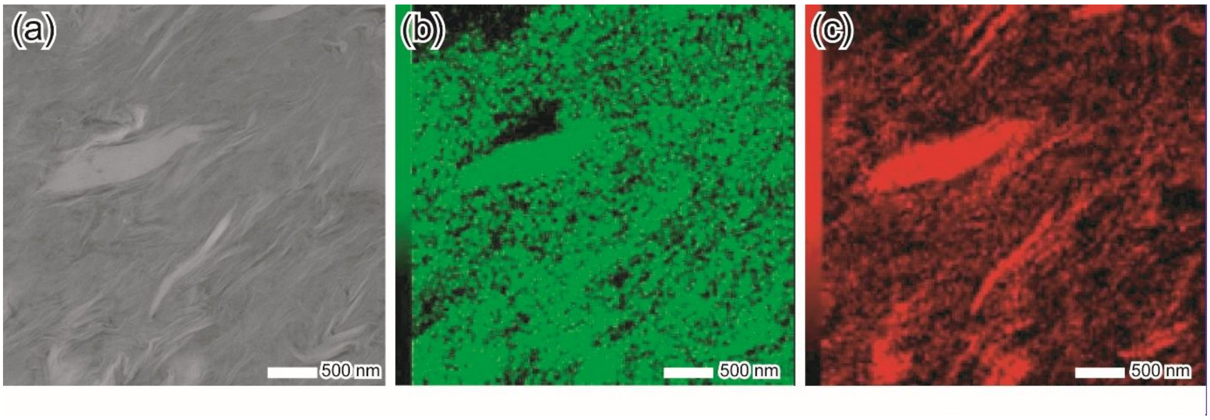

Figure S3. (a) HAADF-STEM image and EDX mapping images of (b) Si and (c) Al of the silica–bentonite composite.

E. Time dependence of R_{sp} value of main component and subcomponent and ratio of subcomponent and main component.

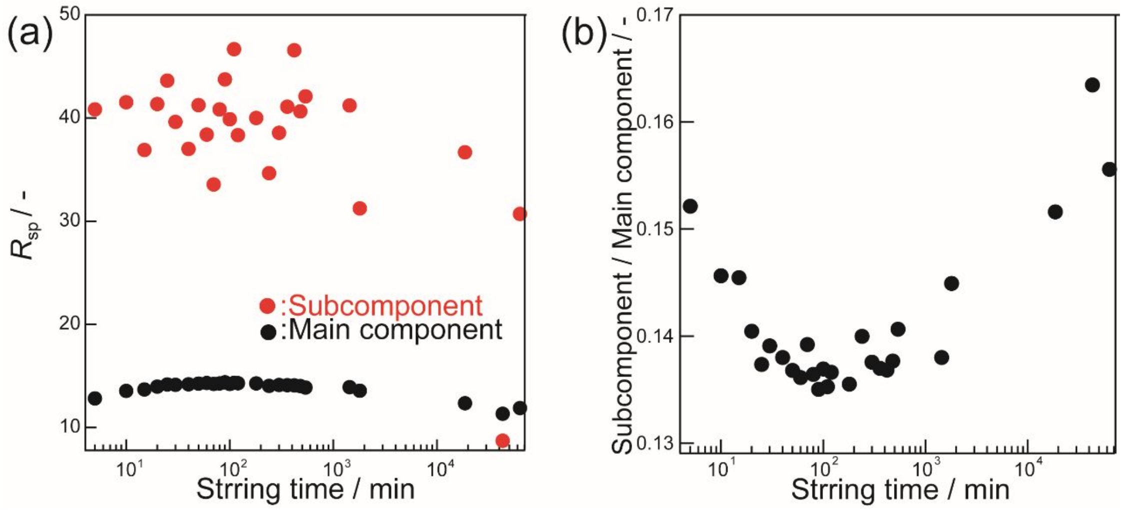

Figure S4. (a) Time dependence of R_{sp} value of main component and subcomponent and (b) ratio of subcomponent and main component.
